# Modern Surgery—General and Operative

**Published:** 1901-10

**Authors:** 


MODERN SURGERY—GENERAL AND OPERATIVE. By John Chalmers DeCosta, M. D., Professor of the Principles of Surgery and of Clinical Surgery, Jefferson Medical College, Philadelphia, Etc. With 493 illustrations. Third edition, revised and enlarged. Pages, 1117. Price, $5.00. Philadelphia and London: W. B. Saunders & Co. 1900.

This is a good practical work on surgery, condensed it is true, but all the facts are given and the irrelevant matter left out. It embraces all the late improvements in both general and operative surgery. The illustrations are modern and appropriate. As a book, the work by DaCosta is as good as any, if not better. T. J. B.

